# Dexmedetomidine Continuous Infusion vs. Remifentanil Target-Controlled Infusion for Conscious Sedation in Otosclerosis Surgery—A Prospective, Single-Center, Randomized Controlled Trial

**DOI:** 10.3390/jcm14092869

**Published:** 2025-04-22

**Authors:** Caius Mihai Breazu, Alma Aurelia Maniu, Ioan Florin Marchis, Matei Florin Negrut, Răzvan Alexandru Ciocan, Florin Vasile Mihăileanu, Violeta Necula

**Affiliations:** 1 1st Department of Anesthesia and Intensive Care, “Iuliu Haţieganu” University of Medicine and Pharmacy, 400347 Cluj Napoca, Romania; csbreazu@yahoo.com; 2 Department of Anesthesia and Intensive Care “Clinicilor 4-6”, Cluj County Emgency Clinical Hospital, 400349 Cluj Napoca, Romania; 3 Research Association in Anesthesia and Intensive Care (ACATI), 400394 Cluj Napoca, Romania; 4 Otorhinolaryngology Department, “Iuliu Hatieganu” University of Medicine and Pharmacy, 400347 Cluj Napoca, Romania; violeta.necula@umfcluj.ro; 5 Faculty of Medicine, “Iuliu Hatieganu” University of Medicine and Pharmacy, 400394 Cluj Napoca, Romania; mateinegrut@gmail.com; 6 Department of Surgery—Practical Abilities, “Iuliu Hațieganu” University of Medicine and Pharmacy, 400337 Cluj Napoca, Romania; razvan.ciocan@umfcluj.ro; 7 Department of Surgery, County Hospital, “Iuliu Hatieganu” University of Medicine and Pharmacy, 400139 Cluj Napoca, Romania; florin.mihaileanu@umfcluj.ro

**Keywords:** otosclerosis, sedation, monitored anesthesia care, dexmedetomidine, remifentanil, surgery, randomized controlled trial

## Abstract

**Background/Objectives:** Otosclerosis causes progressive hearing loss through abnormal bone remodeling within the otic capsule and predominantly affects young individuals. Surgical intervention can markedly enhance a patient’s quality of life and socio-economic status. Anesthetic management may involve either general anesthesia or monitored anesthesia care, with the latter enabling real-time assessment of hearing improvement while providing optimal surgical conditions and patient satisfaction. This study examines the efficacy and safety of continuous dexmedetomidine infusion and target-controlled remifentanil infusion for conscious sedation combined with local anesthesia in otosclerosis surgery. **Methods:** Seventy-four adult patients undergoing otosclerosis surgery were randomly assigned to either the dexmedetomidine group or the remifentanil group. Primary outcomes included patient satisfaction at 24 h post-surgery and surgeon satisfaction with operative conditions. Secondary outcomes comprised hemodynamic effects, the necessity for adjuncts to the proposed sedation protocols, and intra- and postoperative complications. **Results:** There was no statistically significant difference between the dexmedetomidine and remifentanil groups regarding patient satisfaction (*p* = 0.943) and surgeon satisfaction (*p* = 0.069). A strong correlation was observed between surgeons’ assessments and patients’ satisfaction Composite Scores (η^2^ = 0.185, *p* = 0.003). Dexmedetomidine was more effective in significantly reducing arterial pressure and heart rate without undesirable clinical effects. **Conclusions**: No significant difference was found between the groups concerning patient and surgeon satisfaction. Dexmedetomidine infusion led to considerable reductions in arterial pressure and heart rate compared to remifentanil.

## 1. Introduction

Otosclerosis is a condition of the inner ear, with a prevalence of 0.4–1% in the general population, characterized by abnormal bone remodeling within the otic capsule [1]. This process initially causes conductive hearing loss due to stapedo-ovalar ankylosis. In advanced cases, it may progress to mixed or sensorineural hearing loss. The etiopathogenesis is unclear, but studies suggest an autosomal dominant inheritance with incomplete penetrance, affecting young adults, mostly women (4:1 ratio) [1,2]. Surgical treatment involves removing the fixed stapes and placing a prosthetic piston to restore sound transmission from the ossicles to inner ear fluids [3].

The anesthetic management of otosclerosis surgery typically involves either general anesthesia or monitored anesthesia care (MAC) combined with local anesthesia, depending on institutional protocols, the surgeon’s preference, or the patient’s request [4,5,6]. General anesthesia ensures patient immobility and comfort during surgery. General anesthetic techniques include total intravenous anesthesia, which is advocated for better surgical conditions and smoother recovery compared to inhalational anesthesia [7,8]. Both methods benefit from short-acting opioids like remifentanil [8]. Short-acting hypotensive agents, beta blockers, hemostatic agents, and dexmedetomidine further optimize the surgical field [7,9,10]. Airway control is managed with tracheal intubation or supraglottic devices [7,8]. General anesthesia is preferred by many patients and surgeons to prevent local anesthetic pain or unexpected patient movements [6,7]. However, it may lead to side effects such as postoperative nausea and vomiting, prolonged time in the operating room due to anesthesia induction and recovery phases, and more complex postoperative care [8,11]. There are no specific regional anesthesia techniques for middle ear interventions because the ear exhibits heterogeneous sensory innervation, including branches from the cervical plexus and cranial nerves V, VII, IX, and X [5,12]. Local anesthesia involves circular infiltration of the ear canal, and it is essential to combine this with an effective sedation technique to ensure comfort for both the patient and the operator. The objectives of sedation are:

- An immobile operating field;
- Minimal bleeding;
- Hemodynamic and respiratory stability;
- Reducing the risk of postoperative nausea and vomiting;
- Patient comfort.

The significant advantage of conscious sedation over general anesthesia or deep sedation is the possibility of real-time feedback from the patient in case of the sudden onset of vertigo due to an excessively long piston or for hearing testing after fitting the prosthesis [13]. Deep sedation can lead to an uncooperative patient with involuntary movements or upper airway obstruction, potentially compromising the operating field [13,14].

Otosclerosis surgery has been standardized to ensure a brief and consistent procedure with a high success rate. As such, it can be implemented widely as an office intervention or day surgery. To facilitate patient and surgeon comfort and enable a swift discharge, a safe and efficient conscious sedation regimen and rapid recovery process is required [15]. To this point, there is no consensus in the literature favoring a certain drug or sedation technique for this procedure since the publications on the matter are scarce and of low quality.

Dexmedetomidine is a selective alpha-2 adrenergic receptor agonist that appeared in clinical anesthetic practice relatively recently, with sedative effects, providing a reduction in required opioid doses, reduced frequency of delirium and agitation, perioperative sympathicolysis, cardiovascular stabilizing effect, and preservation of respiratory function [10,16,17]. Remifentanil is a synthetic, potent, ultrashort-lived opioid used for postoperative analgesia, sedation, or general anesthesia [18]. Both agents can meet the needs of stapes surgery: a calm and cooperative patient, and reduced bleeding in the surgical field by lowering blood pressure and cardiac output.

The objective of this study was to compare dexmedetomidine (continuous infusion) to remifentanil (target-controlled infusion according to the Minto effect-site model) for MAC in a cohort of patients undergoing otosclerosis surgery. To the best of our knowledge, this is the only trial comparing these two drugs for sedation in otosclerosis surgery.

## 2. Materials and Methods

### 2.1. Study Design and Participants

This study was a prospective, parallel-group, single-blind, randomized controlled trial conducted over 12 months in a tertiary ENT clinic. Adults aged 18 to 65 years presenting for otosclerosis surgery were eligible participants. Patients with an American Society of Anesthesiology (ASA) classification III or higher, requiring general anesthesia, refusing participation, allergic to study medication, with cognitive impairment, or having severe bradycardia or atrioventricular block were excluded.

### 2.2. Randomization and Blinding

Participants were randomly assigned in a 1:1 ratio to either the dexmedetomidine group (group D) or the remifentanil group (group R). Randomization used a computer-generated random sequence. Patients and surgeons were blinded to the group allocation, and those providing postoperative care and collecting postoperative data were also unaware of the group allocation. The anesthesiologists administering the sedative medication were not blinded to group allocation due to the need to closely supervise the study infusion protocols for patients’ safety. They did not participate in assessing the postoperative outcomes.

### 2.3. Intervention

Before surgery, eligible patients were approached by the investigator and invited to participate in the study. The investigator described the study’s features, what to expect during the intervention, and informed the patients that they would complete a satisfaction questionnaire 24 h after surgery. Written informed consent containing relevant information about the trial was provided to the participants. All recruited patients signed the written informed consent.

Upon arrival at the operating room, standard ASA monitoring was provided, including respiratory rate derived from the ECG leads. A peripheral intravenous line was established, and oxygen was administered via nasal cannula with a flow of 5 L/min. Each patient received dexamethasone 4 mg and midazolam 15 microg/kg following the establishment of the intravenous line.

For group D, we administered dexmedetomidine in continuous infusion at a dose of 1 µg/kg over 15 min. Before completing the dexmedetomidine bolus load, 1 µg/kg of fentanyl was given. The patient was checked verbally at regular intervals. We aimed for an Observer’s Assessment of Alertness/Sedation Scale (OAA/S) score of 3 (moderate sedation with eyes closed and responding only to loud verbal stimuli) or 4 (lethargic response to name called in normal tone). A maintenance dose of dexmedetomidine at 0.5 µg/kg/h was continued. After 20 min from the start of the dexmedetomidine loading dose, and approximately 5 min after the fentanyl bolus, local anesthetic infiltration began.

In group R, the continuous infusion commenced similarly, following the administration of 15 µg/kg midazolam and 4 mg dexamethasone. A Minto effect-site model of target-controlled infusion (Agilia SP TIVA, Fresenius Kabi AG, Bad Homburg, Germany) was utilized [19], starting with a concentration of 1 ng/mL and increasing incrementally until a target concentration of 2 ng/mL was reached. If the patient reached an OAA/S score of 3 or their respiratory rate dropped below 8/min before reaching the target concentration of 2 ng/mL, the concentration was not increased further. Conversely, if the desired sedation level (OAA/S score of 3–4 and respiratory rate of 8–10/min) was not achieved, the concentration was incrementally increased above 2 ng/mL until the desired sedation level was reached. Approximately 5–10 min after the start of the remifentanil infusion, the surgeon administered local anesthetic to the ear.

Both groups received local anesthesia with 2% lidocaine and 1/80,000 adrenaline via a 23G needle. The injection began subcutaneously at the crease between the tragus and helix, followed by deeper infiltration to block the tympanic branch of the auriculotemporal nerve. The posterior part of the external auditory canal (EAC) was infiltrated to block facial nerve branches supplying the concha. The EAC was also infiltrated at four cardinal points to block vagus nerve branches, and the tragus region was injected for complete anesthesia. In all cases, an endaural approach was used.

If sedation was unsatisfactory due to patient restlessness, the anesthetist administered a 20–30 mg propofol bolus as needed but stopped ten minutes before piston placement. The surgeon then checked with the patient for hearing, dizziness, or nausea after inserting the piston.

During the intervention, the attending anesthetist closely monitored the patient’s vital signs, with particular attention to airway patency and oxygen saturation. If the oxygen saturation dropped below 94%, the patient was verbally stimulated and visually assessed. If necessary, maneuvers for restoring airway patency or airway control could be implemented at any time.

At the end of the intervention, and at regular intervals in the postoperative period, Paracetamol 1 g, Metamizole 1 g, and Ondansetron 4 mg were administered to manage pain and nausea. The patients were transferred to the ward, where they were instructed to remain mostly flat for the next 24 h in accordance with institutional protocol following otosclerosis surgery. Their vitals were monitored for the next six hours until complete recovery from sedation.

### 2.4. Outcomes’ Measure

The intervention’s impact on patient satisfaction was measured 24 h post-procedure using a modified version of the Iowa Satisfaction with Anesthesia Scale (ISAS), a self-administered questionnaire that evaluates patients’ satisfaction with monitored anesthesia care [20].

We included a total of 8 questions from the original ISAS questionnaire, translated into Romanian in clear language, not using medical jargon, which included 4 “positive” and 4 “negative” questions. If the patient’s agreement with a certain question would imply satisfaction with the anesthesia care provided, the question would be considered “positive”, while the opposite applied to “negative” questions. The following questions were included:

Q1. I felt relaxed.
Q2. I felt pain. *
Q3. I felt safe.
Q4. I threw up or felt like throwing up.*
Q5. I itched. *
Q6. I was too hot or cold. *
Q7. I was satisfied with the anesthesia care.
Q8. I would have the same anesthetic again.
* Negative questions are marked with an asterisk

Patients were asked to choose among 6 answers, noted from −3 to 3, as follows: −3: disagree very much; −2: disagree moderately; −1: disagree slightly; 1: agree slightly; 2: agree moderately; 3: agree very much.

An overall Composite Score was also calculated. After reversing the score for “negative” questions, we calculated the mean score for every question, resulting in a Composite Score, ranging from −3 to 3, where −3 represented complete dissatisfaction and 3 represented complete satisfaction.

The Composite Score constituted the primary outcome of the study, alongside the surgeons’ satisfaction regarding the operatory field and sedation quality, assessed immediately after the procedure using a Visual Analogue Scale (VAS) ranging from 1 (poor) to 2 (moderate), 3 (good), and 4 (excellent).

As secondary outcome measures, the hemodynamic effects of dexmedetomidine were compared with those of remifentanil. Heart rate (HR) and blood pressure (BP) were measured every 5 min and recorded every 10 min during the surgery.

Additionally, the need for adjuncts to the proposed sedation algorithms, such as propofol boluses or variations in remifentanil dosage from the target, was documented.

Complications occurring during surgery or within 24 h post-surgery were recorded, including severe bradycardia or hypotension, oxygen desaturation (<90%), agitation or oversedation (OAA/S < 3), and persistent postoperative nausea and vomiting (PONV).

### 2.5. Statistical Analysis

Statistical analysis was performed using SPSS Statistics v29.0.1 (IBM Corp., Armonk, NY, USA) and GraphPad Prism 8. Continuous variables were reported as mean ± standard deviation (SD) and compared using either Student’s t, if normally distributed, or as median (quartile1; quartile 3) and compared using Mann–Whitney U, if not normally distributed. Normality was tested using the Shapiro–Wilk test, and equality of variances was tested using the Levene’s test. Categorical variables were reported as absolute frequencies and percentages and compared using either chi-square or Fisher’s exact test. The Kruskal–Wallis test with the Bonferroni correction was used for comparing the patients’ Composite Scores among multiple groups (stratified by the surgeons’ grading of operating conditions) and effect size was assessed using η^2^ (η^2^ < 0.01, negligible effect; 0.01 ≤ η^2^ < 0.06, small effect; 0.06 ≤ η^2^ < 0.14, moderate effect; η^2^ ≥ 0.14, large effect). A two-tailed *p* < 0.05 was considered statistically significant.

We performed a power calculation analysis, aiming for a power of 80% and a risk for type I errors of 0.05. Assuming a mean difference in Composite Scores between groups of 0.5 and an SD of 0.75, we needed to include at least 35 patients in each group.

### 2.6. Ethical Considerations

The study protocol was approved by the Institutional Review Board (IRB) of Cluj County Emergency Clinical Hospital with the approval reference number 6155/7 February 2024. Written informed consent was obtained from all participants before enrollment. The study was conducted in accordance with the Declaration of Helsinki and Good Clinical Practice guidelines and was prospectively registered in the ClinicalTrials.org database on 14 February 2024, NCT06283836.

## 3. Results

### 3.1. Study Population

**Figure 1:**
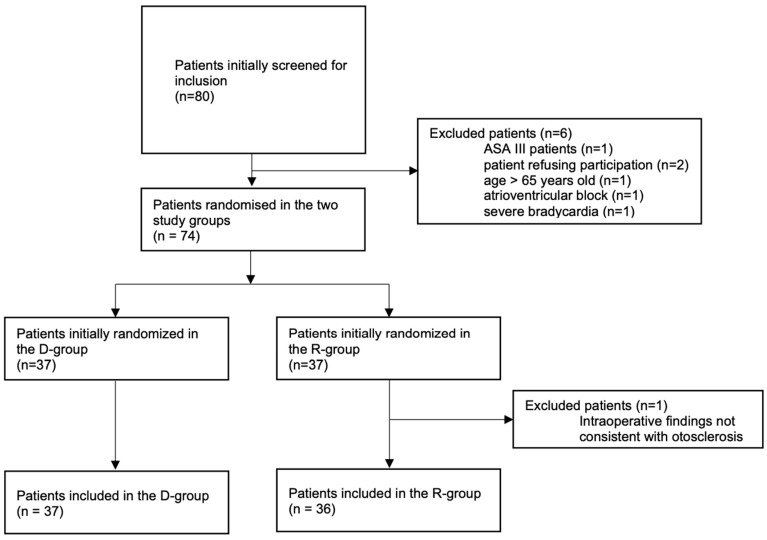
Flow chart of the patients included in the study groups.

Between March 2024 and February 2025, 80 patients were assessed for eligibility. Six were excluded, leaving 74 for randomization. One patient in the remifentanil group was later excluded due to inconsistent intraoperative findings. We analyzed 37 patients in group D and 36 in group R. All 73 remaining patients completed the survey (Figure 1).

Among the 36 patients in the R group, 6 patients required a remifentanil concentration above 2 ng/mL, while 3 patients required less than 2 ng/mL of remifentanil. The remifentanil concentration ranged from 1.1 to 3 ng/mL. The target plasma concentration for patients receiving remifentanil below 2 ng/mL was 1.37 ± 0.23 ng/mL (mean ± standard deviation), and for those requiring concentrations above 2 ng/mL, it was 2.6 ± 0.33 ng/mL. The demographic characteristics were similar between the 2 study groups (Table 1).

### 3.2. Primary Outcomes

A summary of the patients’ responses to the survey questions is provided in Table 2. Overall, the patients’ feedback was mostly positive, with the most common complaint being nausea and vomiting, in 21 cases (28.76%). All patients expressed satisfaction with the anesthesia care and stated they would like to have the same type of MAC in the future

For further analysis, we dichotomized the patients’ answers, considering answers “−3”, “−2”, and “−1” as “disagreement” and answers “1”, “2”, and “3” as “agreement”. The dichotomized answers were then used to compare the satisfaction between the study groups. No statistically significant difference was found in the patients’ answers (Table 3).

**Figure 2:**
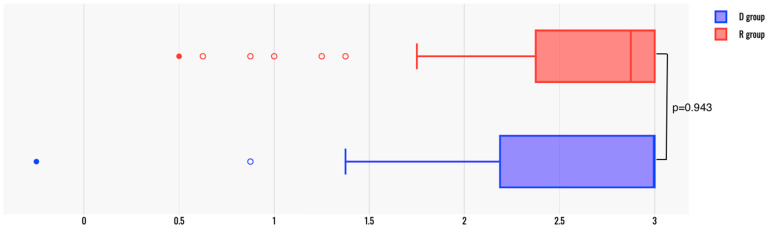
The Composite Score in the two patient groups.

The Composite Scores were comparable between the two groups (median (q1; q3) was 3 (2.25; 3) in group D and 2.88 (2.38; 3) in group R, *p* = 0.943) (Figure 2). Only one patient in the D group had a negative composite score of −0.25, while all patients had positive scores in group R (minimum score was 0.5 in group R). A total of 19 patients (51.35%) in group D had a maximum composite score of 3, while 17 patients (47.22%) in the R group had a maximum score (*p* = 0.724).

**Figure 3:**
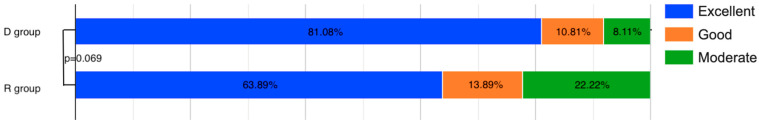
Surgeons’ grading of operatory conditions in the two patient groups.

The surgeons’ satisfaction was also assessed (Figure 3), and no statistically significant difference was found between the surgeons’ grading of operating conditions in the two groups (*p* = 0.069) In none of the cases, the surgical conditions were classified as “poor”.

### 3.3. Secondary Outcomes

Hemodynamic parameters were also compared between the study groups (Table 4). While no difference in the baseline value of systolic, diastolic, and mean blood pressure (BP) or heart rate (HR) was found, the patients in the D group had a lower minimum intraoperative BP and HR than those in the R group. Moreover, patients in the R group had a smaller drop in blood pressure (both as absolute values and as a percent of initial values) and HR (as a percent of baseline, but not as an absolute value) compared to those in the D group. There was no difference between groups in the surgery duration or need for supplementary propofol administration.

**Figure 4:**
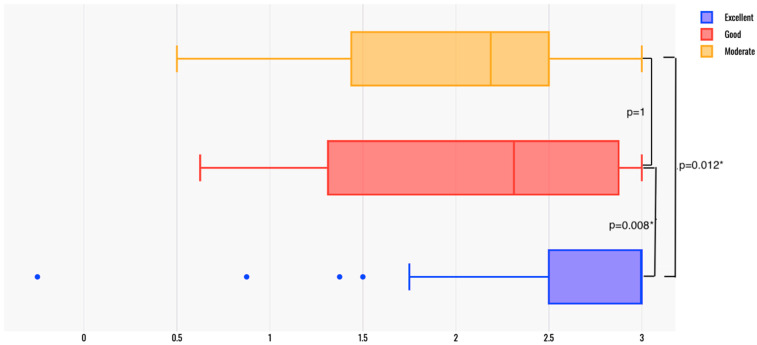
Composite Scores compared between patients grouped by the surgeons’ grading. * Statistically significant difference.

Patient satisfaction scores were compared among patients based on the surgeons’ assessment of operatory conditions (Figure 4). The “excellent” group had a median (q1; q3) Composite Score of 3 (2.5; 3), while the “good” group scored 2.31 (1.34; 2.88) and the “moderate” group scored 2.19 (1.66; 2.44) (*p* < 0.01, Kruskal–Wallis test). Post hoc analysis with the Bonferroni correction indicated a statistically significant difference between the “excellent” and “good” groups (*p* = 0.008) and between the “excellent” and “moderate” groups (*p* = 0.012), whereas no significant difference was observed between the “good” and “moderate” groups (*p* = 1) (Figure 4). There was a strong correlation between the surgeons’ assessments and the patients’ Composite Scores (η^2^ = 0.185, *p* = 0.003).

### 3.4. Complications

None of the patients experienced severe bradycardia necessitating atropine administration during surgery or desaturation requiring supplemental oxygen (other than the nasal cannula provided pre-operatively to all patients) or airway maneuvers. At 24 h postoperatively, none of the patients exhibited severe bradycardia, hypotension, desaturation, agitation or oversedation.

## 4. Discussion

Otosclerosis is a condition that affects young people and causes progressive hearing loss [2]. Stapes surgery does not pose a vital risk, but a successful outcome can positively impact a patient’s life and socio-economic status [3]. Anesthetic management should aim to provide optimal conditions for the surgeon, ensure a quick intervention, and result in patient satisfaction. Monitored anesthesia care (MAC), with or without local anesthetic infiltration or topical application, is an effective method of providing comfort and safety to both patients and surgeons in procedures requiring patient collaboration and involving the head: awake craniotomy with brain mapping, ophthalmic surgery, and ear surgery. The inability to directly access a patient’s airway presents an additional challenge [10,21,22].

In our study, both dexmedetomidine and remifentanil conscious sedation for otosclerosis surgery resulted in high levels of satisfaction among patients and surgeons. Despite a high prevalence of postoperative nausea and vomiting, the majority of patients reported positive satisfaction scores at 24 h. Nausea and vomiting are common complications after stapes surgery, often accompanying vertigo, with an estimated occurrence ranging from 3.4% to 70%, primarily caused by irritation of the membranous labyrinth [23,24].

The surgeons’ satisfaction with the quality of sedation and the surgical field was high, with excellent and good ratings exceeding 90% in group D and 75% in group R, though there was no statistical significance between the groups. We found a statistically significant correlation between the surgeons’ approval of the surgical conditions and the patients’ satisfaction, indicating that patient contentment with anesthetic care translated to good operating conditions.

Monitored anesthesia care (MAC) may contribute to an improved operatory field by lowering arterial pressure and cardiac output and thereby reducing bleeding. Consequently, achieving a degree of hypotension and bradycardia is desirable during stapes surgery and middle ear surgery in general. Our trial demonstrated that dexmedetomidine was more effective than remifentanil in reducing both arterial pressure and heart rate without causing undesired clinical effects.

Several papers compared general anesthesia with MAC and local infiltration for stapes surgery, concluding there are no significant differences in functional outcomes, surgery-related stress, and quality of life [4,25]. Patients appear to cope equally well with the stress of general anesthesia and with undergoing surgery under sedation and local anesthesia. A limited number of studies examined different drugs or combinations of drugs for monitored anesthesia care in middle ear surgery [14,26,27]. Therefore, the ideal sedation regimen for such interventions remains unclear, and practitioners often rely on personal experience rather than standardized protocols provided by the literature.

Sedation and local anesthesia can reduce the duration of procedures, lower risks associated with general anesthesia, and enable the procedure to be carried out in a day care facility or office, thereby reducing overall costs and hospital burdens. Fast recovery and early ambulation may enhance patient satisfaction [28]. Additionally, it offers the advantage of assessing prosthesis positioning during surgery, evaluating hearing improvement, and monitoring labyrinth function in real time [27]. General anesthesia may be necessary for patients with neurological, psychiatric, pulmonary, or orthopedic conditions who cannot tolerate lying flat during surgery. While uncontrolled patient movement can lead to labyrinth injury with serious consequences, no significant differences were reported in the incidences of dead ear between local and general anesthesia [29]. Moreover, only a small percentage of patients report experiencing pain during either the injection of the local anesthetic or the surgery itself [13]. The efficiency of local anesthetic infiltration may vary based on surgeon experience, concentration and type of local anesthetic used, or patient anatomical characteristics. Since only a limited volume of anesthetic can be injected into the restricted space of the external auditory canal due to its cartilaginous nature, the infiltration technique is crucial [13,27,28].

Our study compared two sedation regimens for otosclerosis implemented in our institution in recent years. Ensuring a tranquil patient with reduced bleeding during surgery, while preventing any sudden movements, is essential. Additionally, maintaining an unobstructed airway is crucial, as airway manipulation can compromise the operative field [26]. Therefore, achieving “collaborative sedation” and employing fast-acting aids for uncomfortable moments, such as local anesthesia infiltration, which may be the most painful part of the procedure, is vital [5,21,29]. A single anesthetic agent may not suffice for all stages of stapes surgery using conscious sedation.

The comparison between dexmedetomidine and remifentanil has garnered attention due to their suitability as adjuncts to general anesthesia (for hemodynamic stability intra-operatively, smooth emergence, and postoperative comfort) as well as primary agents for MAC requiring varying levels of sedation. Dexmedetomidine offers unique benefits among sedative agents, including significant intrinsic analgesic effects primarily through reduced sympathetic tone, remarkable respiratory stability, and a calm, cooperative patient [10,16,17]. Given that some studies describe dexmedetomidine as more effective against pain when used as an analgesic-sparing agent, we chose to incorporate a fixed dose of opioid into our sedation protocol to ensure consistent analgesia during local anesthetic infiltration [17].

Dexmedetomidine reaches a consistent effect level approximately 5 to 10 min after bolus dose administration, necessitating the initiation of the infusion at least 20 min prior to local infiltration for optimal sedation [16,30,31]. Adhering to this recommendation may be challenging in a busy operating theatre. The loading dose is critical for short- to medium-duration procedures, as the plateau level of sedation achieved post-bolus endures, mitigating infiltration pain and allowing for intensification if necessary, using short-acting adjuncts like propofol or titrated opioid doses [21]. A potential drawback is prolonged postoperative sedation proportional to the infusion duration, possibly delaying discharge [31,32]. However, in our study, patients instructed to lie flat post-surgery seemed to benefit from residual sedation.

Remifentanil has advantages for collaborative sedation when administered using a target-controlled approach. It demonstrates a rapid clinical onset and is a potent opioid with strong analgesic and hypnotic properties distinct among opioids [18,33]. However, the precise target for achieving the desired level of sedation is not well established [33,34]. We selected a target of 2 ng/mL according to the Minto model effect-site due to its fast onset and relatively safe dose within the lower range of recommendations [33,34]. Despite this, adjustments were necessary for approximately 25% of the patients due to either too deep or too shallow levels of sedation. Importantly, we did not observe an increased incidence of postoperative pain in the remifentanil group despite its short duration of action.

Although the ISAS questionnaire was previously validated as a tool for assessing patient satisfaction with MAC [20], we opted for using a shorter version of the original survey. We excluded 3 questions from the original ISAS (“I felt pain during the surgery”, I hurt”, and “I felt good”), as they overlapped with two of the questions we included (“I felt pain” and “I felt relaxed”), and we considered that the excluded questions would not be relevant when translated into Romanian. A modified version of the ISAS scale was also validated in an Arabic-speaking population, showing good reliability [35].

Our study has certain limitations. It was a single-center trial with a relatively small sample size. The blinding process did not encompass all medical staff involved in patient care. In group D, a small bolus of opioid was used for improved analgesia, while in group R, deviations from the proposed target were made in 25% of participants for safety and comfort reasons. These factors may introduce bias. We assessed sedation depth through clinical judgment alone, without employing objective measures such as the Bispectral Index (BIS). We did not utilize a specific questionnaire for identifying obstructive sleep apnea (OSA) beyond the preanesthetic examination. Furthermore, it is unclear whether the high incidence of postoperative nausea and vomiting was partially attributable to the sedative medication, as the surgery itself can cause vertigo and nausea, and our questionnaire did not specifically address dizziness. Lastly, we did not identify similar studies for comparison.

## 5. Conclusions

The study indicated that both dexmedetomidine and remifentanil are suitable for conscious sedation in stapes surgery, providing high levels of satisfaction to both surgeons and patients. There was no significant difference between the groups in terms of patient and surgeon satisfaction with the procedure, but a strong correlation existed between patient and surgeon satisfaction in both groups. Dexmedetomidine combined with a small fentanyl bolus resulted in a statistically larger reduction in blood pressure and heart rate during the interventions compared to remifentanil.

## Figures and Tables

**Table 1:** Comparison of demographic data of patients in group D and group R.

	All Patients (*n* = 73)	D Group (*n* = 37)	R Group (*n* = 36)	*p*
Gender				0.801
M	13 (17.81)	7 (18.92)	6 (16.67)	
F	60 (82.19)	30 (81.08)	30 (83.33)	
Age	45.9 ± 7.47	45.14 ± 8.18	46.69 ± 6.68	0.376
BMI (kg/m^2^)	25.1 ± 4.05	25.29 ± 4.26	24.9 ± 3.88	0.878
ASA				0.926
I	45 (61.64)	23 (62.16)	22 (61.11)	
II	28 (38.36)	14 (37.84)	14 (38.89)	

Abbreviations: M, masculine; F, feminine; BMI, body mass index; ASA, American Society of Anesthesiologists.

**Table 2:** Patients’ responses to the questionnaire.

Questions	Groups	Grade					
		−3	−2	−1	1	2	3
I felt relaxed.	D	0 (0)	0 (0)	0 (0)	0 (0)	6 (16.22)	31 (83.78)
	R	0 (0)	1 (2.78)	0 (0)	2 (5.56)	5 (13.89)	28 (77.78)
I felt pain. *	D	28 (75.68)	0 (0)	0 (0)	2 (5.41)	2 (5.41)	5 (13.51)
	R	23 (63.89)	4 (11.11)	0 (0)	4 (11.11)	3 (8.33)	2 (5.56)
I felt safe.	D	0 (0)	0 (0)	0 (0)	0 (0)	5 (13.51)	32 (86.49)
	R	0 (0)	0 (0)	0 (0)	0 (0)	1 (2.78)	35 (97.22)
I threw up or felt like throwing up. *	D	26 (70.27)	0 (0)	0 (0)	5 (13.51)	1 (2.7)	5 (13.51)
	R	25 (69.44)	1 (2.78)	0 (0)	2 (5.56)	1 (2.78)	7 (19.44)
I itched. *	D	33 (89.19)	0 (0)	1 (2.7)	1 (2.7)	1 (2.7)	1 (2.7)
	R	35 (97.22)	0 (0)	1 (2.78)	0 (0)	0 (0)	0 (0)
I was too hot or cold. *	D	34 (91.89)	0 (0)	0 (0)	2 (5.41)	0 (0)	1 (2.7)
	R	29 (80.56)	1 (2.78)	1 (2.78)	2 (5.56)	1 (2.78)	2 (5.56)
I was satisfied with the anesthesia care.	D	0 (0)	0 (0)	0 (0)	0 (0)	3 (8.11)	34 (91.89)
	R	0 (0)	0 (0)	0 (0)	0 (0)	1 (2.78)	35 (97.22)
I would have the same anesthetic again.	D	0 (0)	0 (0)	0 (0)	0 (0)	3 (8.11)	34 (91.89)
	R	0 (0)	0 (0)	0 (0)	1 (2.78)	3 (8.33)	32 (88.89)

* Negative questions are marked by an asterisk, and answers are shown before reversing the scores for negative questions.

**Table 3:** Comparison of the responses of patients in the D group and R group after dichotomization.

		All Patients (*n* = 73)	D Group (*n* = 37)	R Group (*n* = 36)	*p*
I felt relaxed.	disagree	1 (1.37)	0 (0)	1 (2.78)	0.493
	agree	72 (98.63)	37 (100)	35 (97.22)	
I felt pain. *	disagree	55 (75.34)	28 (75.68)	27 (75)	0.947
	agree	18 (24.66)	9 (24.32)	9 (25)	
I felt safe.	disagree	0 (0)	0 (0)	0 (0)	1
	agree	73 (100)	37 (100)	36 (100)	
I threw up or felt like throwing up. *	disagree	52 (71.23)	26 (70.27)	26 (72.22)	0.854
	agree	21 (28.77)	11 (29.73)	10 (27.78)	
I itched. *	disagree	70 (95.89)	37 (100)	33 (91.67)	0.115
	agree	3 (4.11)	0 (0)	3 (8.33)	
I was too hot or cold. *	disagree	65 (89.04)	34 (91.89)	31 (86.11)	0.479
	agree	8 (10.96)	3 (8.11)	5 (13.89)	
I was satisfied with the anesthesia care.	disagree	0 (0)	0 (0)	0 (0)	1
	agree	73 (100)	37 (100)	36 (100)	
I would have the same anesthetic again.	disagree	0 (0)	0 (0)	0 (0)	1
	agree	73 (100)	37 (100)	36 (100)	

* Negative questions are marked by an asterisk, and answers are shown before reversing the scores for negative questions.

**Table 4:** Hemodynamic data and medication administered in the two groups.

	All Patients (*n* = 73)	D Group (*n* = 37)	R Group (*n* = 36)	*p*
SBPi (mmHg)	130.49 ± 15.26	130.94 ± 14.85	129.97 ± 15.96	0.895
DBPi (mmHg)	72.63 ± 10.08	72.86 ± 11.35	72.35 ± 8.55	0.836
MBPi (mmHg)	91.92 ± 10.68	92.22 ± 11.24	91.56 ± 10.16	0.802
HRi (bpm)	78.6 ± 11.99	76.5 ± 11.12	81.03 ± 12.66	0.124
SBPm (mmHg)	104.19 ± 11.5	99.61 ± 8.16	109.52 ± 12.6	<0.001
DBPm (mmHg)	58.52 ± 8.2	55.69 ± 6.32	61.81 ± 8.96	<0.001
MBPm (mmHg)	73.75 ± 8.74	70.33 ± 6.39	77.71 ± 9.5	00.002
HRm (bpm)	62.6 ± 9.34	59.08 ± 6.42	66.68 ± 10.57	0.001
SBP drop (mmHg)	26.3 ± 13.65	31.33 ± 12.38	20.45 ± 12.86	<0.001
SBP drop (% of SBPi)	19.64 ± 8.47	23.41 ± 6.86	15.26 ± 8.12	<0.001
DBP drop (mmHg)	14.52 ± 9.31	17.17 ± 9.13	11.45 ± 8.68	0.008
DBP drop (% of SBPi)	19.22 ± 10.6	22.6 ± 9.56	15.3 ± 10.54	0.004
MBP drop (mmHg)	18.17 ± 10.17	21.89 ± 9.09	13.85 ± 9.75	<0.001
MBP drop (% of MBPi)	19.25 ± 9.22	23.15 ± 7.39	14.71 ± 9.16	<0.001
HR drop (bpm)	16.9 ± 8.32	18.64 ± 8.53	14.87 ± 7.71	0.085
HR drop (% of HRi)	21.15 ± 9.07	23.96 ± 8.99	17.88 ± 8.14	0.005
Propofol administration				0.781
yes	11 (15.07)	6 (16.22)	5 (13.89)	
no	62 (84.93)	31 (83.78)	31 (86.11)	
Propofol dose (mg)	31.82 ± 15.37	26.67 ± 13.66	38 ± 16.43	0.242
Propofol dose (mg/kg)	0.49 ± 0.27	0.41 ± 0.27	0.59 ± 0.25	0.277
Atropine administration				1
yes	0 (0)	0 (0)	0 (0)	
no	73 (100)	37 (100)	36 (100)	
Surgery duration (min)	61.47 ± 18.87	60.81 ± 20.12	62.26 ± 17.55	0.623

Abbreviations: SBPi, initial systolic blood pressure; DBPi, initial diastolic blood pressure; MBPi, initial mean blood pressure; HRi, initial heart rate; SBPm, minimal systolic blood pressure; DBPm, minimal diastolic blood pressure; MBPm, minimal mean blood pressure; HRm, minimal heart rate.

## Data Availability

Data are fully available upon request to the corresponding author.

## References

[1] Uppal S., Bajaj Y., Rustom I., Coatesworth A.P. (2009). Otosclerosis 1: The Aetiopathogenesis of Otosclerosis. Int. J. Clin. Pract..

[2] Cureoglu S., Schachern P.A., Ferlito A., Rinaldo A., Tsuprun V., Paparella M.M. (2006). Otosclerosis: Etiopathogenesis and Histopathology. Am. J. Otolaryngol..

[3] Kari E., Wilkinson E.P., Kountakis S.E. (2013). Stapedectomy and Stapedotomy. Encyclopedia of Otolaryngology, Head and Neck Surgery.

[4] Wegner I., Bittermann A.J.N., Zinsmeester M.M., Van Der Heijden G.J.M., Grolman W. (2013). Local versus General Anesthesia in Stapes Surgery for Otosclerosis: A Systematic Review of the Evidence. Otolaryngol. Head Neck Surg..

[5] Bakhos D., Rouf C.-E., Laffont M., Lescanne E. (2021). Stapes Surgery for Otosclerosis under Local Anaesthesia with Sedation. Eur. Ann. Otorhinolaryngol. Head. Neck Dis..

[6] Liu Y.F., Gupta A., Nguyen S.A., Lambert P.R., Jung T.T. (2020). Preferences in Stapes Surgery among American Otological Society Otologists. World J. Otorhinolaryngol. Head Neck Surg..

[7] Pairaudeau C., Mendonca C. (2019). Anaesthesia for major middle ear surgery. BJA Educ..

[8] Mukherjee K., Seavell C., Rawlings E., Weiss A. (2003). A comparison of total intravenous with balanced anaesthesia for middle ear surgery: Effects on postoperative nausea and vomiting, pain and conditions of surgery. Anaesthesia.

[9] Zhang Y., Han L., Ding W., Gao L., Feng Y., An H. (2024). Intravenous tranexamic acid significantly improved visualization and shortened the operation time in microscopic middle ear surgery: A randomized controlled trial. Int. J. Surg..

[10] Munhall C.C., Warner B.K., Nguyen S.A., Guldan G.J., Meyer T.A. (2023). Use of dexmedetomidine for controlled hypotension in middle ear surgery: A systematic review and meta-analysis. Am. J. Otolaryngol..

[11] Watcha M.F. (2002). Postoperative Nausea and Emesis. Anesthesiol. Clin. N. Am..

[12] Peuker E.T., Filler T.J. (2002). The Nerve Supply of the Human Auricle. Clin. Anat..

[13] Lavy J., Powell H. (2013). Stapes Surgery under Local Anaesthesia. Ann. R. Coll. Surg. Engl..

[14] Nallam S., Chiruvella S., Reddy A. (2017). Monitored Anaesthesia Care–Comparison of Nalbuphine/Dexmedetomidine versus Nalbuphine/Propofol for Middle Ear Surgeries: A Double-Blind Randomised Trial. Indian J. Anaesth..

[15] Voizard B., Maniakas A., Saliba I. (2019). Office-Based Stapes Surgery. Otolaryngol. Head Neck Surg..

[16] Kaur M., Singh P. (2011). Current Role of Dexmedetomidine in Clinical Anesthesia and Intensive Care. Anesth. Essays Res..

[17] Bhana N., Goa K.L., McClellan K.J. (2000). Dexmedetomidine. Drugs.

[18] Beers R., Camporesi E. (2004). Remifentanil Update: Clinical Science and Utility. CNS Drugs.

[19] Minto C.F., Schnider T.W., Gregg K.M., Henthorn T.K., Shafer S.L. (2003). Using the Time of Maximum Effect Site Concentration to Combine Pharmacokinetics and Pharmacodynamics. Anesthesiology.

[20] Dexter F., Aker J., Wright W.A. (1997). Development of a Measure of Patient Satisfaction with Monitored Anesthesia Care: The Iowa Satisfaction with Anesthesia Scale. Anesthesiology.

[21] Goettel N., Bharadwaj S., Venkatraghavan L., Mehta J., Bernstein M., Manninen P.H. (2016). Dexmedetomidine vs. Propofol-Remifentanil Conscious Sedation for Awake Craniotomy: A Prospective Randomized Controlled Trial. Br. J. Anaesth..

[22] Kaya C., Celebi N.O., Debbag S., Canbay O., Onal O. (2022). Comparison of Dexmedetomidine and Remifentanil Infusion in Geriatric Patients Undergoing Outpatient Cataract Surgery: A Prospective, Randomized, and Blinded Study. Med. Gas. Res..

[23] Necula V., Maniu A.A., Ujváry L.-P., Dindelegan M.-G., Tănase M., Tănase M., Blebea C.M. (2023). Vertigo Associated with Otosclerosis and Stapes Surgery—A Narrative Review. Medicina.

[24] Harmat K., Thurén G., Simon L., Nepp N., Németh A., Gerlinger I., Bakó P. (2017). Posztoperatív vertigo vizsgálata stapedotomián és stapedectomián átesett betegeknél. Orvosi Hetil..

[25] Luryi A.L., Schettino A., Bojrab D.I., Babu S.C., Michaelides E.M., Bojrab D.I., Schutt C.A. (2021). Hearing Outcomes and Complications in Stapes Surgery for Otosclerosis Performed Under General or Local Anesthesia. Otolaryngol. Head Neck Surg..

[26] Riou J.B., Rouf C.E., Moriniere S., Bakhos D., Lescanne E. (2021). Otosclerosis Surgery under Local Anesthesia with Sedation: Assessment of Quality of Life and Stress. Eur. Ann. Otorhinolaryngol. Head. Neck Dis..

[27] Giannoni B., Pollastri F., Adembri C., Straticò D., Vannucchi P., Stival A., Checcucci C., Bruno C., Pecci R. (2022). Hearing Outcomes and Patient Satisfaction after Stapes Surgery: Local versus General Anaesthesia. Acta Otorhinolaryngol. Ital..

[28] Lazard D.S., Donné F., Lecanu J.B. (2019). Day-Surgery in Otology: Impact Study of a Dedicated Organizational Model. Eur. Ann. Otorhinolaryngol. Head. Neck Dis..

[29] Vital V., Konstantinidis I., Vital I., Triaridis S. (2008). Minimizing the Dead Ear in Otosclerosis Surgery. Auris Nasus Larynx.

[30] Schnabel A., Meyer-Frieem C.H., Reichl S.U., Zahn P.K., Pogatzki-Zahn E.M. (2013). Is Intraoperative Dexmedetomidine a New Option for Postoperative Pain Treatment? A Meta-Analysis of Randomized Controlled Trials. Pain.

[31] Sim J.H., Yu H.J., Kim S.T. (2014). The Effects of Different Loading Doses of Dexmedetomidine on Sedation. Korean J. Anesthesiol..

[32] Koo J.M., Chung Y.-J., Lee M., Moon Y.E. (2023). Efficacy of Dexmedetomidine vs. Remifentanil for Postoperative Analgesia and Opioid-Related Side Effects after Gynecological Laparoscopy: A Prospective Randomized Controlled Trial. JCM.

[33] Eleveld D.J., Colin P., Absalom A.R., Struys M.M.R.F. (2020). Target-Controlled-Infusion Models for Remifentanil Dosing Consistent with Approved Recommendations. Br. J. Anaesth..

[34] Motamed M.C., Roubineau M.R., Depoix M.J.-P., Servin M.F., Roche C.G., Billard M.V. (2021). Efficacy of Target Controlled Infusion of Remifentanil with Spontaneous Ventilation for Procedural Sedation and Analgesia (Remi TCI PSA): A Double Center Prospective Observational Study. J. Opioid Manag..

[35] Baroudi D.N., Nofal W.H., Ahmad N.A. (2010). Patient Satisfaction in Anesthesia: A Modified Iowa Satisfaction in Anesthesia Scale. Anesth. Essays Res..

